# NGS-guided precision oncology in metastatic breast and gynecological cancer: first experiences at the CCC Munich LMU

**DOI:** 10.1007/s00404-020-05881-z

**Published:** 2020-12-04

**Authors:** Elena Sultova, C. Benedikt Westphalen, Andreas Jung, Joerg Kumbrink, Thomas Kirchner, Doris Mayr, Martina Rudelius, Steffen Ormanns, Volker Heinemann, Klaus H. Metzeler, Philipp A. Greif, Alexander Burges, Fabian Trillsch, Sven Mahner, Nadia Harbeck, Rachel Wuerstlein

**Affiliations:** 1 Department of Obstetrics and Gynecology and CCC Munich LMU University Hospital, Ludwig Maximilians University (LMU), Munich, Germany; 2 Department of Internal Medicine III and CCC Munich LMU, University Hospital, Ludwig Maximilians University (LMU), Munich, Germany; 3 Institute of Pathology and CCC Munich LMU, University Hospital, Ludwig Maximilians University (LMU), Munich, Germany; 4 Gynecologic Oncology Center and CCC Munich LMU University Hospital, Ludwig Maximilians University (LMU), Munich, Germany; 5 Breast Center and CCC Munich LMU University Hospital, Ludwig Maximilians University (LMU), Munich, Germany

**Keywords:** Personalized medicine, Breast cancer, Ovarian cancer, Biomarker, Molecular diagnostic, Molecular tumor board

## Abstract

**Purpose:**

Comprehensive genomic profiling identifying actionable molecular alterations aims to enable personalized treatment for cancer patients. The purpose of this analysis was to retrospectively assess the impact of personalized recommendations made by a multidisciplinary tumor board (MTB) on the outcome of patients with breast or gynecological cancers, who had progressed under standard treatment. Here, first experiences of our Comprehensive Cancer Center Molecular Tumor Board are reported.

**Methods:**

All patients were part of a prospective local registry. 95 patients diagnosed with metastatic breast cancer or gynecological malignancies underwent extended molecular profiling. From May 2017 through March 2019, the MTB reviewed all clinical cases considering tumor profile and evaluated molecular alterations regarding further diagnostic and therapeutic recommendations.

**Results:**

95 patients with metastatic breast or gynecological cancers were discussed in the MTB (68% breast cancer, 20% ovarian cancer, 5% cervical cancer, 3% endometrial cancer and 4% others). Genes with highest mutation rate were PIK3CA and ERBB2. Overall, 34 patients (36%) received a biomarker-based targeted therapy recommendation. Therapeutic recommendations were implemented in nine cases; four patients experienced clinical benefit with a partial response or disease stabilization lasting over 4 months.

**Conclusion:**

In the setting of a multidisciplinary molecular tumor board, a small but clinically meaningful group of breast and gynecological cancer patients benefits from comprehensive genomic profiling. Broad and successful implementation of precision medicine is complicated by patient referral at late stage disease and limited access to targeted agents and early clinical trials.

**Trial registration number:**

284-10 (03.05.2018).

## Introduction

In women, metastatic breast cancer and gynecological malignancies are among the most frequent causes of cancer death. In 2018, there were an estimated 2,088,849 new cases of breast cancer and 626,679 deaths, 569,847 new cases of cervical cancer and 311 365 deaths, and 295,414 new cases of ovarian cancer and 184,799 deaths worldwide. [1] Despite rising overall incidence, mortality rate has steadily decreased owing to early detection and improvements in the therapeutic management of these patients. However, although the development of new drugs, vaccines, and systematic screening programs has improved patients’ outcomes, effective measures to successfully treat metastatic cancer are still missing.

With the advent of molecular diagnostics, cancer treatment entered a new era. New techniques of sequencing DNA such as comprehensive genomic profiling (CGP) and hotspot next generation sequencing (NGS) provide tools for deciphering complete genes and later entire genomes at unprecedented speed [2]. These new approaches led to the development of a novel cancer treatment movement, known as precision medicine. By selecting the most effective treatment based on the molecular characteristics of tumor tissues or some other biologic parameters of the malignant disease, precision medicine aims to offer personalized treatment concepts to cancer patients with limited standard of care options. Molecular therapeutic agents (MTA) targeting individual actionable molecular alterations have been successfully developed in the past few years, showing the positive impact of using molecular-based therapy on the cancer patients’ outcome [3–6]. These include the use of growth factor receptor 2 antibody trastuzumab in breast cancer, a tyrosine kinase inhibitor imatinib in myelogenous leukemia associated with the BCR-ABL fusion gene and EGFR tyrosine kinase inhibitors in lung carcinomas [7, 8].

**Fig. 1:**
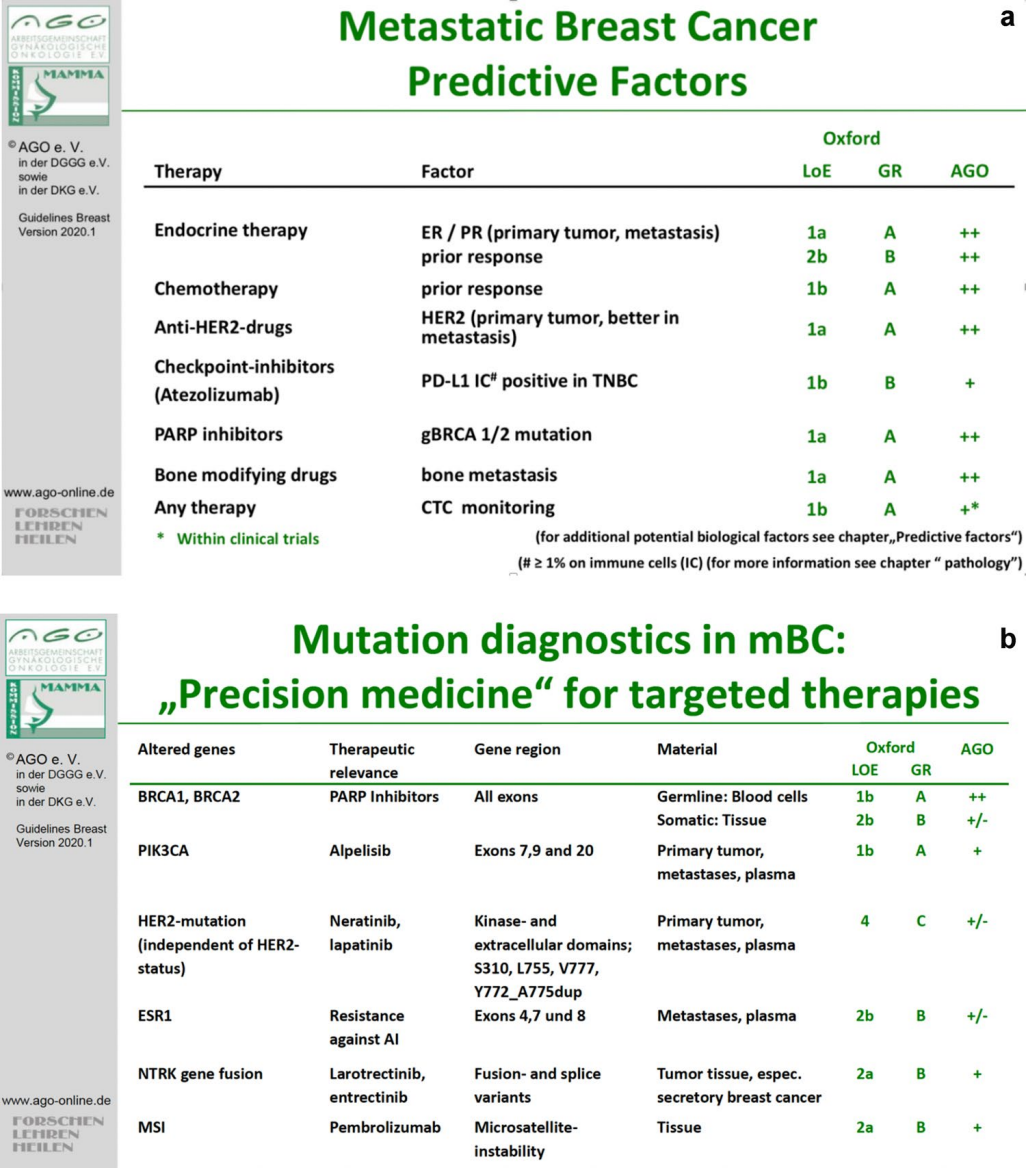
Predictive factors (**a**) and treatment-relevant genetic alterations (**b**) in metastatic breast cancer, German Gynecological Oncology Group. In 2018, AGO was the first international guideline-commission to make recommendations regarding precision medicine in breast cancer. (http://www.ago-online.de) [14]

Breast and gynecological cancers constitute a heterogeneous group of malignant diseases associated with multiple genetic alterations [9–11]. In the past few years, a growing number of molecular markers in breast cancer, for example, have been investigated and some of them are now well-established as reliable predictors of prognosis and response to tumor therapy (Fig. 1a). Moreover, many different targeted therapies have been approved for use in breast cancer treatment (Fig. 1b). The recent approval of the PIK3CA specific inhibitor alpelisib has been the most recent example of targeted agents moving into routine care. [12] Treatment with alpelisib was shown to prolong PFS by more than 6 months compared to the control arm. [13]

In gynecologic malignancies, MTAs have also been successfully implemented into clinical care. For example, early data from a clinical phase II trial focusing on BRCA-mutated ovarian cancer showed that olaparib as maintenance treatment significantly improved progression-free survival (PFS) in relapsed platinum-sensitive ovarian cancer [15]. In 2018, these data could be transferred to the first line setting when treatment effects of the SOLO1 trial were presented [16]. Due to an impressive PFS improvement and a 70% lower risk of disease progression or death with olaparib compared to placebo, this effect led to the incorporation of PARP inhibitors into the primary treatment of ovarian cancer in 2019 [17]. However, when it comes to other gynecologic malignancies such as endometrial cancer, the development of MTA is delayed in comparison to other malignancies.

By detecting potential actionable pathways using molecular diagnostics, it is also possible to assess and treat various cancer types. For example, the ERBB2/PIK3/AKT/mTOR pathway is known for its relevance in breast cancer, but recently a relevant actionable mutation from the same pathway, PIK3R1^W624R^ was also identified in ovarian cancer [18]. Another study suggested that some subtypes of cervical cancers may also benefit from existing ERBB2/PIK3/AKT/mTOR targeted agents [19].

With the rising number of MTAs and considering the heterogeneous molecular profiles of breast cancer and gynecological malignancies, it is reasonable to expect that patients with these malignancies could potentially benefit from implementation of precision oncology based on comprehensive genomic profiling (CGP) into clinical care. Promising early data for such malignancies has been presented in multiple trials. In breast cancer, many reports of such driver alterations have emerged in the past few years, suggesting that patients could profit from precision medicine and targeted therapies [20]. For example, in the SAFIR01 multicenter prospective trial, data of precision medicine benefitting breast cancer patients were presented. 9 out of 43 patients (21%) responded to the recommended targeted therapy with a stable disease lasting over 16 weeks [21]. In ovarian cancer, multiplatform molecular profiling, conducted in a commercially available profiling center, led to a significantly longer post-profiling survival in patients, who were treated with profile-guided targeted agents, in comparison to the control group [22].

With the technical advances in molecular diagnostics and the continuous approval of many targeted therapies, the growing field of precision medicine is constantly expanding and requires optimization. Considering the complexity of precision medicine in oncology, it was reasonable to create a molecular tumor board (MTB) to leverage the knowledge of the many different disciplines involved in oncological treatment and to provide optimal treatment recommendations. In this manuscript, first experiences of the Comprehensive Cancer Center (CCC) LMU Munich Molecular Tumor Board are presented.

The aim of this project was to retrospectively measure the impact of MTB discussions and recommendations made by a multidisciplinary tumor board on outcome of patients with breast and gynecological cancers progressing under standard treatment. Detailed information including data on patient characteristics, diagnostic and treatment recommendations, implementation of the recommendations, and outcome of treated patients with breast and gynecological cancers (ovarian, endometrial, cervix, and other type of cancer) are presented.

## Materials and methods

**Fig. 2:**
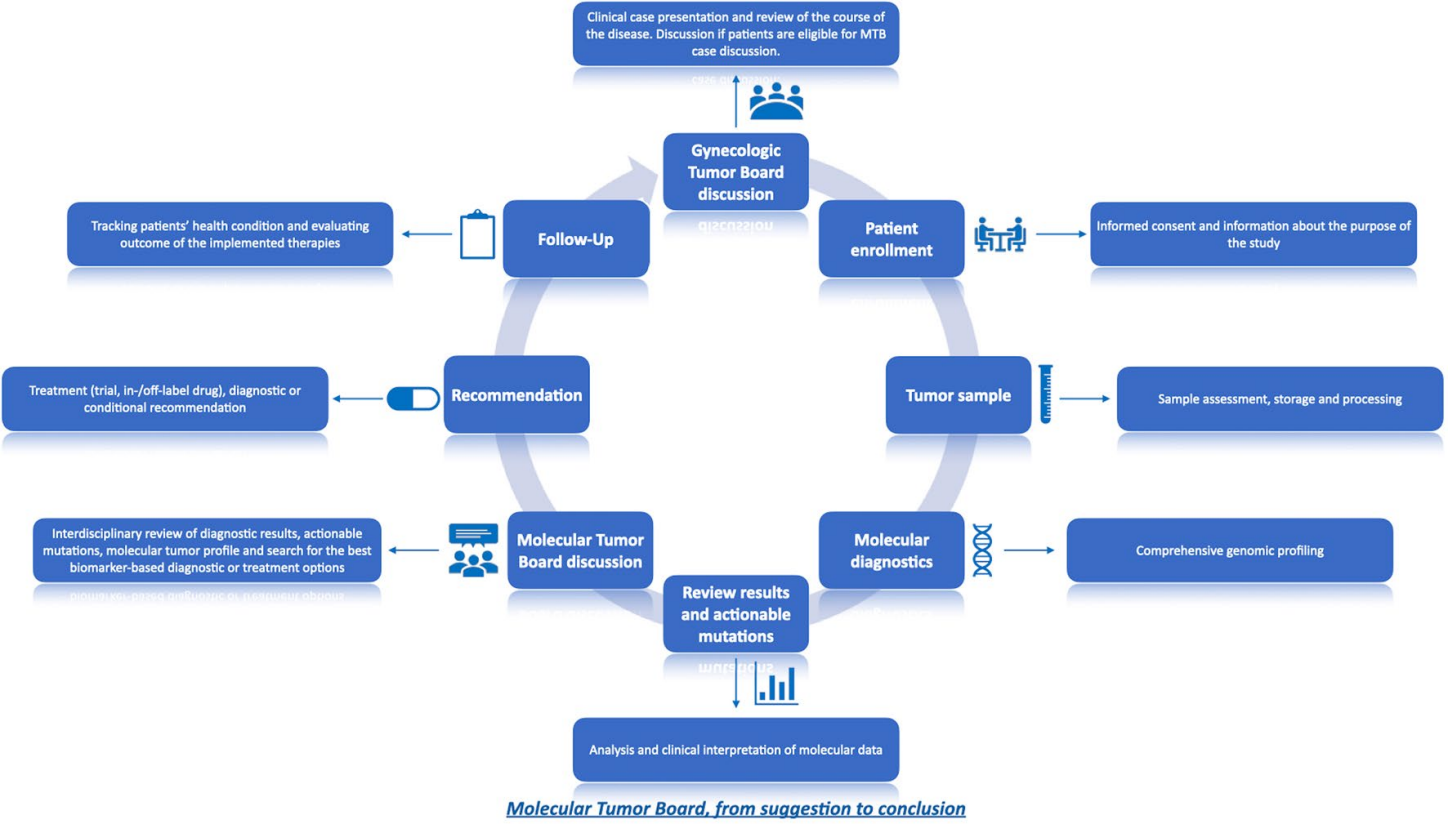
MTB, from suggestion to conclusion

All patients reported here were discussed in the local MTB, which reviewed clinical cases and the respective tumor profiles with the associated actionable alterations. The final result of each MTB case discussion was a report, focused on NGS data and diagnostic and potential diagnostic, and therapeutic alternatives. Thereby, the MTB presented itself as a multidisciplinary team (MDT), which comprised clinical oncologists, pathologists, molecular pathologists, genetic counselors, bioinformaticians, and scientists with expertise in genetic and tumor profiling in diverse cancers. MTB-meetings were held every 2 weeks with the purpose of interpretation and/or translation of the molecular diagnostics’ results into diagnostic and/or treatment recommendations. All patients’ cases were first presented at organ-specific gynecology tumor boards by a team of experienced gyneco-oncologists, who reviewed all the clinical course of every individual patient and discussed if patients were eligible for a MTB discussion. Apart from recent tumor material, recent radiology images and other diagnostic tests were also required for the interdisciplinary setting of the MTB. All treatment recommendations were supported by levels of evidence by using the ESMO Scale for Clinical Actionability of molecular Targets (ESCAT). The process from enrolling the patient into the study till receiving a recommendation by the MTB is shown in Fig. 2.

### Patients and patient informed consent

All patients discussed (*n* = 95) were included in the prospective single-center case study, “The informative Patient”, launched in March 2017 at the LMU University Hospital, Munich as a Munich-site part of the DKTK (German Cancer Consortium) program. All enrolled patients suffered from metastatic breast or gynecological cancer which had progressed after at least one line of prior standard treatment and who had no longer access to curative treatment. Prior to inclusion, all participants signed an informed consent that they were informed about potential and limitations that molecular diagnostics could offer for treatment selection and for analysis of their data, further discussion of their case by a multidisciplinary MTB, as well as for collecting follow-up data on the course of disease for research purpose (including requesting patient data from other physicians and institutions).

The intention-to-treat (ITT) population consisted of 100 patients. Eventually, five patients were excluded, because of death prior to a treatment recommendation or withdrawal of consent.

The data here are based on the results of an ITT population of 95 patients.

### Molecular pathology

Molecular analyses were performed at the Institute of Pathology of the LMU. Appropriate tissue regions were selected histo-morphologically from formalin-fixed paraffin embedded (FFPE)- or fresh frozen tissue. Moreover, liquid biopsies (blood, liquor) were included. In only four patients, analysis had to be repeated due to material constraints. Targeted NGS was performed with the Oncomine Comprehensive Cancer v.3 Panels (Agilent) thereby screening for changes in 161 genes on DNA (SNV, MNV, small ins, del, indels, CNV) and RNA (gene fusions) level. DNA and RNA were isolated using Qiagen's GeneRead DNA FFPE- or RNeasy FFPE-kits, respectively. Nucleic acids (NA; DNA, and RNA) from liquid biopsies were prepared by utilization of the QIAamp Circulating Nucleic Acid Kit. Subsequently, library preparation as first step of NGS was generated by employing Ampliseq Library Plus-, Ampliseq cDNA synthesis-, Ampliseq CD index, Ampliseq Equalizer- together with Ampliseq Comprehensive v3-kits (all Illumina) or DNA- and RNA-Oncomine Comprehensive Panels v3 and Ion AmpliSeq Library-, IonXpress Barcode Adapter-, Ion Library Equalizer-kits together with Ion Chip kits (mostly 550) (all Thermo Fisher), following for each step the respective user manuals. Libraries were run on an Ion Torrent GeneStudio S5 Primer (Thermo Fisher) or Illumina 500 Next Seq (Illumina) NGS machine. Analysis of results was performed with either the Ion-Reporter System (Thermo Fisher) followed by further variant and quality interpretation with a self-made excel tool or annotating VCF-files using wAnnovar (http://wannovar.wglab.org/) [23] together with the self-made python-script PathoMine filtering for clinically relevant mutations. Mutations were judged as relevant on the basis of the key 'interpretation' given in ClinVar [24]. Alterations were confirmed with the Integrated Genomics Viewer (IGV, Broad Institute). The resulting molecular pathological dataset together with data from immunohistochemistry, fluorescence in situ hybridization (FISH), and histo-morphology became part of a comprehensive pathological report which was sent out to the MTB.

### Data assessment

For this analysis, electronic medical records were reviewed for patient characteristics and follow-up. If needed, medical oncologists, gynecologists, and general practitioners were contacted in order to collect follow-up data on treatment course and patient status. Patient characteristics were summarized using descriptive statistics. Follow-up of clinical outcomes was performed to track tumor response to recommended therapies and analyzed by measuring progression-free survival (PFS) of patients, who received the recommended treatment. PFS was calculated from the first day of treatment with the recommended in- or off-label targeted drug until the date of disease progression or death, whichever occurred first, analogous to the Johns Hopkins MTB study and to the Von Hoff et al. study [25]. In order to evaluate the benefit of the treatment recommendation, we then calculated the PFS ratio (PFSr) by comparing the PFS of the recommended treatment and the PFS of the previous therapy of the patients. Cut-off date for data analysis was August 1st, 2019.

## Results

### Patient characteristics

From March 2017 through March 2019, a total of 95 cases were submitted to the MTB. All patients (*n* = 95) were females, had an underlying malignant condition, suffered from metastatic disease, and had experienced disease progression under standard treatment. Patients with implemented therapy recommendations had received a median of five (range 2–6) prior therapies for metastatic cancer. The median age at time of the initial MTB presentation was 52 years (range 19–82 years).

**Fig. 3:**
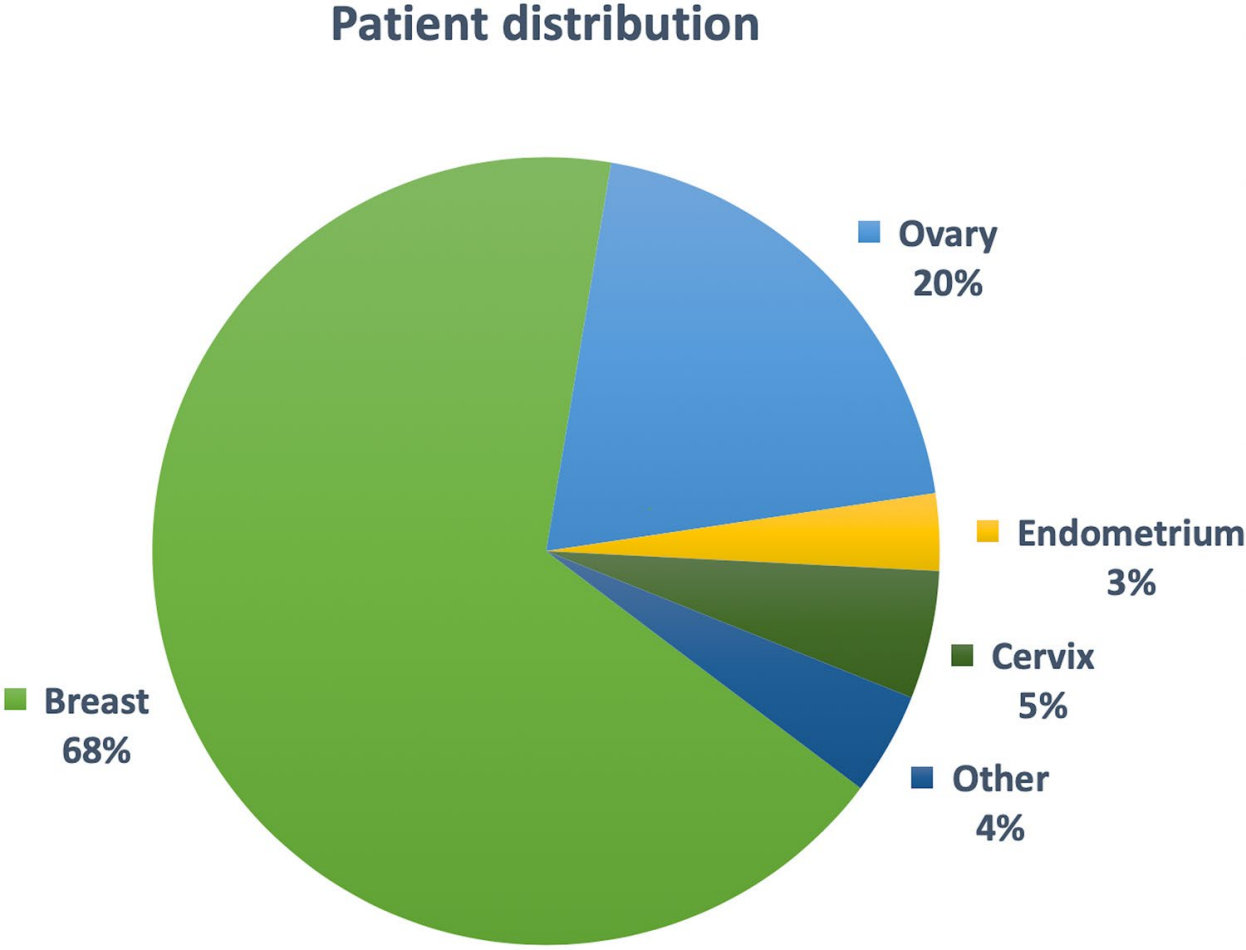
Distribution of the cases discussed at the MTB meeting by tumor entity (*n* = 95)

As shown in Fig. 3, the most frequent tumor type was breast cancer (*n* = 64, 68%), followed by ovarian cancer (*n* = 19, 20%). The majority of patients with breast cancer had triple-negative (ER, PR and HER2 negative; *n* = 30; 46.9%), followed by estrogen receptor (ER) -positive and/or progesterone receptor (PR) -positive, human epidermal growth factor receptor 2 (HER2) -negative (luminal-like) (n = 28; 43.8%), or HER2 positive, ER-negative, PR-negative disease (n = 5; 7.8%) at the time of the MTB case discussion; one patient (1.6%) had triple-positive disease (ER positive and/or PR positive, HER2 positive).

Characteristics of patients with a molecular profile are reported in Table 1.

**Table 1:** Patient characteristics

Covariables	
Median age at diagnosis	47 years (range 12–80)
Age at diagnosis	
< 30	5 (5.3%)
30–39	27 (28.4%)
40–49	21 (22.1%)
50–59	30 (31.6%)
60–69	8 (8.4%)
≥ 70	4 (4.2%)
Median age at MTB case presentation	52 years (range 19–82)
Age at MTB case presentation	
< 30	2 (2.1%)
30–39	19 (20.0%)
40–49	20 (21.1%)
50–59	28 (29.5%)
60–69	18 (18.9%)
≥ 70	8 (8.4%)

### Molecular profiling

Molecular tests using NGS were performed for all 95 patients. Out of the set of mutations from the molecular pathological NGS-analysis, actionable mutations were defined as those matching or informing the use of available targeted agents.

Four patients had tumor sequencing performed twice during the course of disease. 81 (85.3%) patients had suitable tissues for multimodal molecular profiling (NGS). All in all, 103 molecular alterations were identified in 55 cases (57.9%). The median number of alterations observed in each sample was one (range 0–6). Out of the 55 patients, 41 (43.2%) had an actionable mutation, which the board reviewed as a potentially targetable. No genomic alterations in the 161 investigated genes were found in 40 (42.1%) analyses, in 14 (14.7%) of which the molecular diagnostics test was technically not successful because of poor DNA quality or insufficient material quality. Although five (5.3%) patients had an actionable mutation, they did not receive a therapy recommendation because of co-morbidities, not meeting trial inclusion criteria, or other requirements for receiving a specific targeted therapy.

**Fig. 4:**
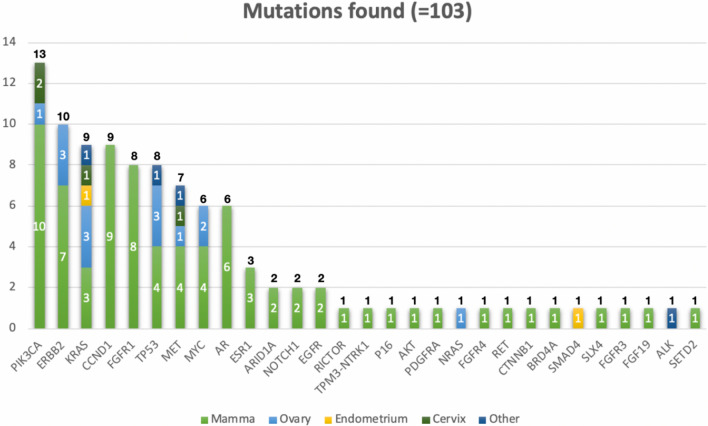
Frequency of genomic alterations for the different tumor entities (*n* = 95)

We discovered mutations in over 30 different genes. Among the patients tested, the most common alterations were as follows: PIK3CA mutation (13/95; 13.7%); ERBB2 mutation (10/95; 10.5%); KRAS mutation (9/95; 9.5%), and CCND1 mutation (9/95; 9.5%). Incidences of genomic alterations by gene and the distribution of molecular alterations by tumor type are shown in Fig. 4.

### Recommendations

Among the 55 (57.9%) patients with at least one molecular alteration identified, 41 patients (43.2%) had an actionable alteration, whereas 14 (14.7%) had only non-actionable variants. Eventually, this resulted in 15 diagnostic and 49 treatment recommendations for 45 patients (47.4%). Multiple recommendations were adjusted for 20 (21.1%) patients (multiple recommendation principle). Six patients received a conditional recommendation, which required specific further diagnostics, two of which resulted in a treatment recommendation.

### Diagnostic recommendations

Out of 15 diagnostic recommendations, 10 were pursued. In seven (7.4%) cases, extended genetic analyses were recommended and eventually six (6.3%) of them were performed. Re-biopsies were recommended in 14 cases, when the initial diagnostic tests were technically not successful, which we did not include in the evaluation of the final results.

### Therapeutic recommendations

**Fig. 5:**
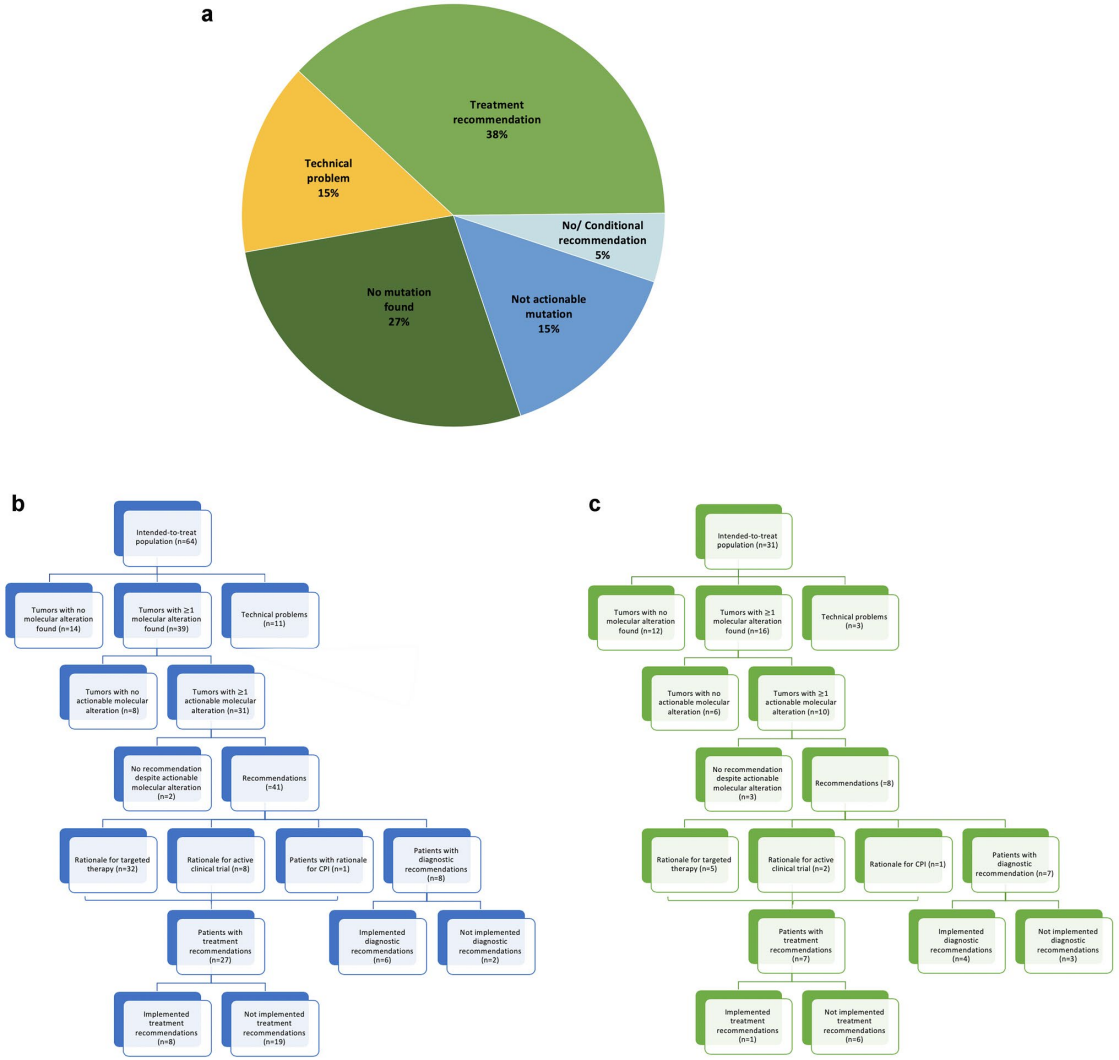
Treatment or diagnostic recommendations. Note, all numbers do not add up because some patients are counted in more than one category (e.g., had an actionable alteration for a treatment recommendation and also for diagnostic recommendation or received more than one treatment/ diagnostic recommendation). **a** Diagram representing the outcome of the molecular diagnostic testing (*n* = 95). **b** Breast cancer patients. **c** Gynecological cancer patients

As shown in Fig. 5, 36 (37.9%) patients were given a therapy recommendation, 14 (14.7%) of whom received more than one treatment suggestion, as their tumor molecular profile revealed more than one actionable mutation. Two (2.1%) patients were excluded from the evaluation of the clinical outcome, as they received the recommended therapy in the period between NGS analysis and MTB treatment recommendation.

Overall, 9 of 34 therapeutic recommendations were pursued. Of note, in the present cohort, no patient pursued the recommended enrollment in a clinical trial. In-label therapy recommendations were implemented in five cases, whereas off-label recommendations were implemented in four patients. The most common reasons for non-administration of MTB-recommended therapy were deterioration of patients’ physical health condition, early death, no access to the recommended drug therapy, declined reimbursement applications by payer, or patient decision (see Table 2).

**Table 2:** Recommendations (Note, some patients received more than one diagnostic and/or treatment recommendation.)

	BC	GC
Patients with min. 1 recommendation	No	No
Diagnostic	8	7
Therapeutic	27	7
No treatment recommendation	30	20
Conditional recommendation	3	3
Referral to organ board		1
Diagnostic recommendations		
Extended genetic analysis	3	4
PD-L1 Test	2	
HR-Status	1	1
Other	5	3
Patients with diagnostic recommendations (*n* = 15)		
Implemented	6	4
Non-implemented	2	3
Treatment recommendations		
Targeted therapy	32	5
Trial inclusion	8	2
Checkpoint inhibition	1	1
Patients with treatment recommendations (*n* = 36)		
Implemented	7	1
Non-implemented	22	6

### Clinical outcome

All patients were included in the registry after multiple standard of care treatments.

**Fig. 6:**
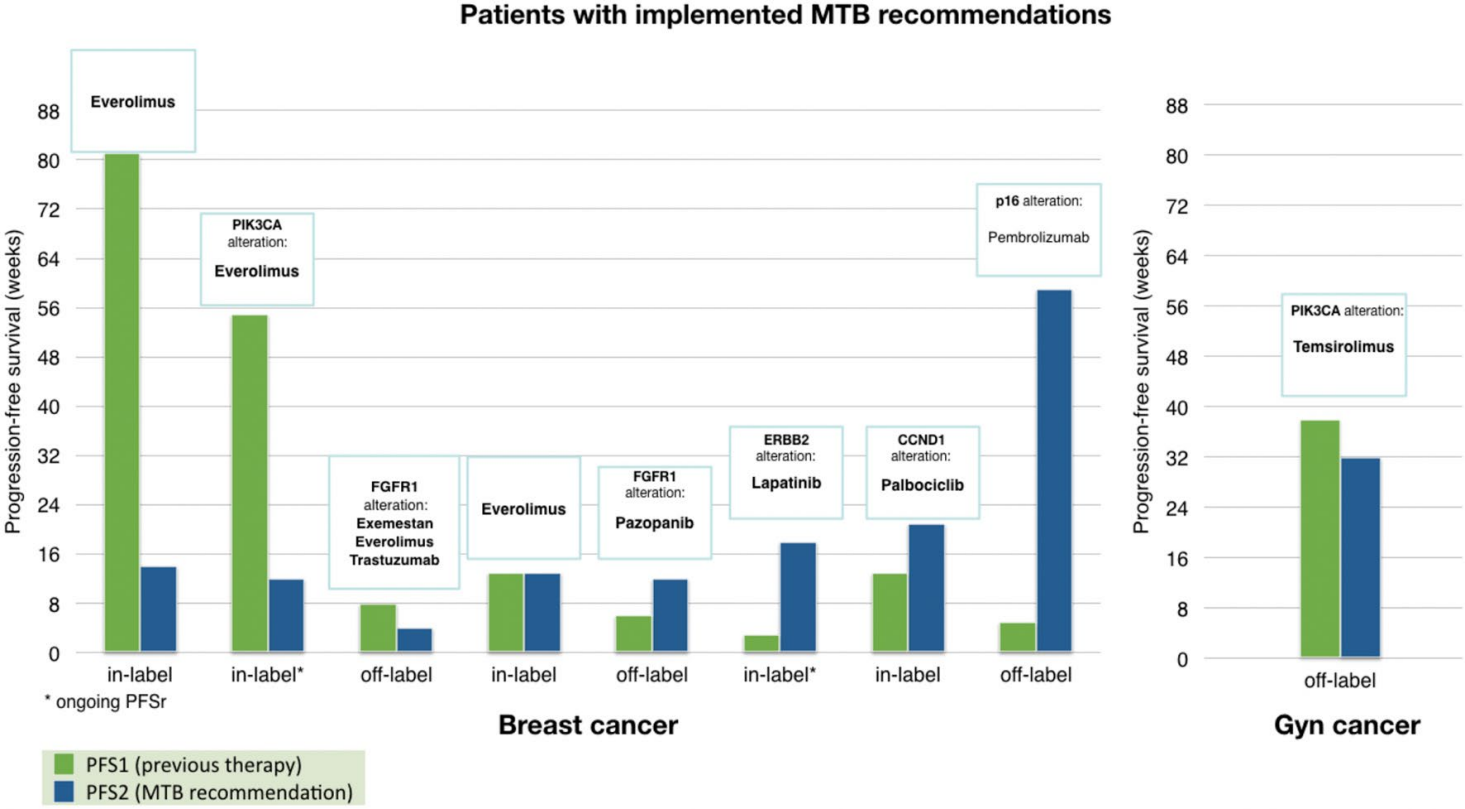
Comparison of PFS of previous line of therapy (PFS1) and implemented therapy recommendation (PFS2). *PFS* the period of time between the start of treatment till disease progression/ death

Out of nine (9.5%) patients following therapy recommendation, 4 (4.2%) showed a state of partial remission or stabilization lasting more than 16 weeks, including two of them receiving off-label therapy recommendation. Comparing PFS of the recommended therapy with the PFS of the previously received systemic treatment, we estimated that four of nine responders receiving MTB-recommended therapies displayed a progression-free survival (PFS) ratio (PFS2/PFS1; PFSr) > 1.3, showing the relevance of the suggested therapies. Two patients responded with an ongoing PFSr. Figure 6 details the actual comparison of PFS on implemented recommended treatment versus PFS on the patient’s last prior treatment.

More information about the outcome of responding patients is shown in Table 3.

**Table 3:** PFS ratio (PFSr) = ratio of patients’ PFS on the implemented recommended therapy (PFS2) (in this case the recommended in- or off-label targeted drug) to their PFS on the most recent previous line of therapy (standard of care) (PFS1)

#	Tumor entity	Treatment	Label	PFS2 (weeks)	PFS1 (weeks)	PFSr
1	Breast	Everolimus	In	14	81	0.17
2	Breast	Everolimus	In	12	55	0.22
3	Breast	Exemestan + Everolimus + Trastuzumab	Off	4	8	0.50
4	Breast	Everolimus	In	13	13	1.00
5	Breast	Pazopanib	Off	12	6	2.00
6	Breast	Lapatinib	In	18	3	6.00
7	Breast	Palbociclib	In	21	13	1.62
8	Breast	Pembrolizumab	Off	59	5	11.80
9	Cervix	Temsirolimus	Off	32	38	0.84

*PFSr* PFS2/PFS1

See Appendix for details of identified actionable mutations and corresponded treatment recommendations made by the MTB.

## Discussion

We evaluated the clinical consequences of actionable genetic alterations (by NGS) in 95 patients with metastatic breast cancer and gynecological malignancies, part of a pilot monocentric patient registry with the purpose of generating real-world data. Forty-one patients (43.2%) had at least one actionable molecular aberration. The total number of patients with a drug-targetable alteration was 34 (35.7%). Overall, 9 of 34 patients (9.5% of all) received the recommended drug treatment. In a small, but significant group of patients, four out of nine with implemented therapy recommendations (44.4%) experienced a clinical benefit (PFSr > 1.3) lasting over 16 months, a result similar to the one shown by Jameson et al. in cases of patients with metastatic breast cancer, who received personalized therapy recommendations based on multi-omic molecular profiling [26, 27].

Precision medicine offers not only personalized treatment concepts for patients, but also helps us optimize diagnostic and treatment options by identifying biomarkers that are linked to response and resistance to immunotherapy. For instance, in the past few years, the problem of resistance to endocrine therapy has been a point of research. Recently, the key role of the acquisition of ligand-independent ESR1 mutation in breast cancer as a common mechanism of resistance to hormonal therapy was discovered [28].

So far, the precision medicine movement is controversial and has sparked multiple debates. On the one hand, the SHIVA trial (2015), one of the first randomized investigation of precision therapy, was negative for its primary endpoint (progression-free survival [PFS]), as no statistically significant difference in PFS between patients receiving molecularly targeted agents and the control arm was demonstrated [29]. On the other hand, studies recruiting large number of patients, such as MOSCATO 01 (2017) and ProfiLER (2017), suggested that high-throughput genomic analyses (i.e. next-generation sequencing, comprehensive genomic profiling) improve clinical outcome in patients with advanced cancers. However, this approach has only been proven to be beneficial to a small subset of patients so far [30, 31]. As shown in Table 4, studies focusing on precision medicine show different, contradictory results. While in some studies more than 20% of the enrolled patients received the recommended according to molecular profiling treatment, in others the number of patients treated remains very low. These results suggest the need for large data collections in order to improve selection criteria and identify markers that discriminate patients that might benefit most from precision medicine.

**Table 4:** Overview of studies focusing on molecular profiling

Author/Study	Tumor entity	Enrolled patients (*n* )	MP patients	Actionable alterations	Implemented therapies—*n* (% of enrolled)	Results
Le Tourneau et al. (SHIVA) [29]	Solid tumors	741	496 (67%)	293 (40%)	96 (13%)	No significant difference in PFS (PFS: 2.3 vs 2.0 *p* = .41), hazard ratio for death or disease progression, 0.88 (95% CI 0.65–1.19)
Stockley et al. (IMPACT/COMPACT) [45]	Solid tumors	1893	1640 (87%)	187 (10%)	84 (5%)	ORR: 19% in genotype-matched group vs 9% in unmatched group, *p* = 0.61
Massard et al. (MOSCATO-01) [30]	Solid tumors	1035	843 (81%)	411 (40%)	199 (24%)	ORR: 11%, SD 52%, PFSr > 1.3: 63/193 (33% of all treated patients or 7% of all enrolled patients)
Trédan et al. (PROFILER) [31]	Solid tumors	2579	1980 (77%)	1032 (40%)	163 (6%)	ORR: 0.9% of all patients
Rodon et al. (WINTHER) [46]	Solid tumors	303	303 (100%)	25 (89%)	107 (35%)	PFSr > 1.5: 22% of the patients with MP-based treatment
Hoefflin et al. [47]	Solid tumors	198	n.a	104 (53%)	33 (17%)	PR: 11/33 (33.3% of all treated patients or 5.5% of all enrolled patients) SD: 8/33 (24.2% of all treated patients or 4% of all enrolled patients)
André et al. (SAFIR01/UNICANCER) [21]	Breast cancer	423	299 (71%)	195 (46%)	55 (13%)	ORR:4 patients had a partial response and 9 had SD > 16 weeks (3% of all patients)
Parker et al. [27]	Breast cancer	43	43 (100%)	40 (93%)	17 (40%)	7 patients (41% of all treated patients or 16% of all enrolled patients) achieved SD or PR

*MP* molecular profiled, *PFS* progression-free survival, *ORR* overall response rate, *SD* stable disease, *PR* disease progression, *n.a.* not available

Although molecular targeted agents themselves are more precise than standard cytotoxic agents, clinical evidence for a significant better outcome associated with MTAs is still missing, as the access to targeted therapies remains limited, making collecting data regarding their efficacy difficult. In order to achieve their implementation in clinical care, a re-assessment of the standards of evidence sufficient to prove the benefit of precision cancer therapies is needed [32]. New evidence suggests that appropriately conducted real-world data studies have the potential to support regulatory decisions in the absence of RCT data [33].

Based on initial results of the CCC LMU Munich, patients of various tumor entities benefit from extended molecular diagnostics and their implementation in clinical care [34]. Recently, many studies have described the positive effect of MTB case discussions for particular groups of patients with advanced solid cancers. However, there is not enough evidence for the utility of MTB decisions for patients with breast and gynecological malignancies.

The world of precision medicine is constantly evolving, and new targeted therapies are being developed and approved, enabling more and more patients (with up to this point of time not actionable mutation) to receive targeted therapies. For example, in spring 2019, the Food and Drug Administration of the USA (FDA) approved the PIK3CA inhibitor alpelisib in combination with endocrine therapy for patients with HR-positive, HER2-negative, PIK3CA-mutated, advanced or metastatic breast cancer. The availability of this drug after start of the Managed Access Program in our clinic could have resulted in five further therapy recommendations in our MTB cohort, showing the need of identifying such alterations in cancer patients.

The rising number of active targetable mutations affects the complexity of the results, making their interpretation a challenge for many oncologists. In 2014, Gray et al. conducted a study, which evaluated cancer physicians’ ability of using multiplex tumor genomic testing and showed that many physicians lack confidence in interpreting complex genomic test results as well as in incorporating them into practice [35]. Thus, we see great potential in establishing the combination of molecular diagnostic tests and a subsequent case discussion by a multidisciplinary molecular board team not only as a routine for cancer patients but also as a training platform and a knowledge-expanding approach for oncologists to help guide their decisions.

However, precision oncology faces some challenges, which delay its widespread translation into clinical practice. Critics of the incorporation of NGS and similar methods into clinical practice express following concerns:

First, the significant cost of molecular diagnostics and targeted drugs is still a great disadvantage. While prices of next-generation sequencing technologies are dropping from about $3 billion in the year 2000 and to $5000 today, the selection of molecular targeted agents is still enormously expensive [36]. As the price of precision medicine is still rather high for most patients, it is now crucial to also evaluate its cost-effectiveness in order to support its translation into clinical practice, for example in the setting of clinical trials and research programs [37].

Second, logistical problems causing limited access to targeted drugs and clinical trials for biomarker-positive patients represent another major problem. This is mainly due to the absence of reimbursement for drugs beyond their labelled indication. As a consequence, in order to receive the required, often off-label drug, patients need to be enrolled within active clinical trials or are required to cover the costs themselves or to file an application for reimbursement by the competent health insurance prior to treatment initiation. Clinical trials often have strict inclusion criteria and are, therefore, not easily accessible to many patients. As shown in the SAFIR01 trial, only a small number of patients benefit from personalized therapies mostly due to drug access problems. This problem could be solved by establishing a portfolio of early phase clinical basket trials or by early-access-programs [38]. Recent studies suggest that the implementation of a MTB improves access to targeted therapy [39]. As seen in our clinic, the early-access-program that we started in November 2019 enabled many patients with a PIK3CA mutation to derive benefit from the targeted drug alpelisib soon after its FDA approval in spring 2019 [40].

Third, another major limitation is the testing of tumors from patients with late stage disease, which limits treatment options and hinders patients from receiving the recommended therapy or from enrolling in a clinical trial. As patients in an advanced cancer situation are often in an unstable health condition, obtaining biopsy material with a good quality of tissue is quite difficult. Our study had 14 (14.7%) technically unsuccessful molecular diagnostics. Moreover, the time between enrolling patients in the study, processing tumor samples, followed by the molecular diagnostics and the MTB case discussion is still rather lengthy in view of the fact that malignancies in late stages tend to evolve at unprecedented speed, while causing deterioration of the general condition and hindering patients from receiving particular therapies, one of the main reasons for the relative low number of implemented therapies (9 out of 34). In this study, molecular profiling and discussion were completed in a clinically reasonable time frame of approximately 4 weeks, which is comparable to the median turnaround times in other studies. Therefore, it is reasonable to expect that introducing molecular profiling at an earlier time point in a patient’s disease trajectory could improve the quality of molecular diagnostics and allow patients to benefit more from a multidisciplinary tailored MTB-based treatment advice.

Fourth, another concern is that the current trend of identifying single variables and matching it with an appropriate targeted therapy may be irrelevant for some patients because of the heterogeneous landscape of their cancer. Disease variability among individual tumors causes patients with tumors of similar histology to respond differently to targeted therapies [41–43]. For example, only 60% of lung cancer patients with the p.L858R mutation in the epidermal growth factor receptor gene (EGFR) respond to gefitinib, although all of them are carriers of the exact same mutation in the target gene, indicating that other, yet unknown genetic aberrations may influence the effect of targeted drugs and that the disease course is still unpredictable to a great extent [44].

Fifth, the common use of medicines outside the approved label is controversial. Off-label drug use may represent a danger for patient safety in some cases, but it is sometimes justified from a clinical point of view. Four out of nine (44%) of the implemented recommended therapies in the study “The informative Patient” included off-label drugs; two of these patients (50%) experienced a clinical benefit with a partial response or stabilization lasting over 4 months, while having progressed under last standard treatment.

There were several limitations to our study. First, despite a relatively high number of breast and gynecological cancer, the overall number of included patients remains low. Second, our patient cohort presented had a heterogeneous tumor type, making general conclusions relatively difficult. Third, the number of patients with implemented therapies is limited, due to deterioration of patients’ general condition or no access to the recommended targeted drug, as previously reported in other studies. Nevertheless, we do demonstrate feasibility of and patient benefit from a routine MTB at a large comprehensive cancer center.

## Conclusion

The landscape of molecular alterations in breast and gynecological cancers is heterogeneous. Advances in the quality and availability of molecular diagnostics and the number of targeted therapies increase rapidly, offering patients with advanced cancer a variety of new treatment options. MTBs try to bridge the gap in between molecular alterations and matching drugs in a structured manner.

The primary objective of the present monocentric study was to estimate, in a real-world setting, the impact of interdisciplinary MTB case discussions for patients with breast and gynecological malignancies. Altogether, on the basis of individual molecular diagnostics, diagnostic and treatment recommendations were made for 45 patients (47.4% of all). Nine out of 34 patients received the recommended treatment. Four out of 9 patients responded with a PFSr > 1.3. Therefore, our results support the approach of matching specific drugs (in- and off-label) to particular genetic aberrations and demonstrate its relevance in breast and gynecological cancers for a small, but clinically relevant group of patients. By providing a multidisciplinary tailored-based treatment advice based on genetic tests, it is now possible for more patients with breast and gynecological malignancies to gain maximum clinical benefit and improve survival of patients with either advanced stage cancer or a rare tumor entity by applying personalized medicine.

The MTB strategy, however, needs to be standardized and optimized in order to eliminate major logistical problems such as limited access to targeted agents (often off-label) and clinical trials, as well as patient referral at stage disease that are too late for a beneficial therapeutic intervention.

## Appendix

See Table

**Table 5:** Data supplement

#	Mutation	Tumor entity	Treatment recommended in MTB	Followed treatment / Line of therapy	PFS (months) after start of treatment
1	FGFR1, androgen receptor and CCND1 amplifications	Breast	1. CDK4/6 Inhibitor 2. Everolimus 3. androgen receptor blocker		
2	CCND1 amplification	Breast	1. CDK4/6 Inhibitor 2. Palbociclib + Fulvestrant 3. Everolimus	Palbociclib	21
3	ERBB2 mutation	Breast	Afatinib / Neratinib		
4	PTEN deletion; MET mutation	Breast	1. NCT03337724 trial 2. Exemestan + Everolimus		
5	PIK3CA mutation	Breast	Everolimus		
6	MET Exon 14 mutation	Breast	Crizotinib		
7	MYC, FGFR1 and CCND1 amplifications	Breast	Everolimus	Everolimus	13
8	androgen receptor amplification	Breast	1. NCT01945775 / NCT02163694 trial 2. Bicalutamide / Tamoxifen		
9	PIK3CA mutation	Breast	1. SOLAR-1 / IPATunity130 trial 2. Everolimus		
10	ERBB2 amplification	Breast	Lapatinib, Trastuzumab, Emtansine and Pertuzumab		
11	ARID1A and PIK3CA mutations, LMB (4,16 muts/MB)	Breast	Everolimus	Everolimus	12
12	ESR1 mutation, CCND1 amplification	Breast	Fulvestrant + Everolimus		
13	TP53 and NOTCH1 mutations	Breast	Cyclophosphamid		
14	TPM3(7)—NTRK1(10) gene fusion	Breast	NCT02568267 trial		
15	MET Exon 2 mutation	Breast	Cabozantinib		
16	KRAS and 2 PIK3CA mutations	Breast	lipos. Doxorubicin / Bevacizumab + Temsirolimus/ Everolimus		
17	androgen receptor mutation, PIK3CA mutation	Breast	Everolimus		
18	FGFR1, CCND1, EGFR, PIK3CA and PDGFRA amplifications	Breast	Pazopanib		
19	ESR1 and PIK3CA mutations	Breast	1. NCT03056755 trial 2. Everolimus		
20	p16 high expression and MYC mutation	Breast	Checkpoint inhibitor	Pembrolizumab	59
21	androgen receptor amplification	Breast	Androgen receptor blocker		
22	AKT mutation	Breast	1. AKT inhibitors 2. IPATunity130 trial 3. Everolimus		
23	SLX4 and TP53 mutations; amplifications: FGFR1, CCND1, FGF19, FGFR3	Breast	Pazopanib	Pazopanib	12
24	ESR1 mutation	Breast	Fulvestrant + CDK4/6 Inhibitoren		
25	CCND1 and FGFR1 amplifications	Breast	1. Everolimus + antihormonal therapy; 2. Dovitinib		
26	PIK3CA and ERBB2 mutations, high expression ERBB2	Breast	1. Pertuzumab/ Trastuzumab (+ Everolimus) 2. Neratinib	Lapatinib	18
27	FGFR1 amplification	Breast	antihormonal therapy + Everolimus + Trastuzumab	Exemestan + Everolimus + Trastuzumab	4
28	CCND1 amplification	Breast	1. Exemestan + Everolimus; 2. NCT-MASTER / TOP-ART trial		
29	CCND1 and FGFR1 amplifications	Breast	1. Everolimus + Exemestan 2. NCT03517956 trial	Everolimus + Exemestan	14
30	KRAS and ERBB2 mutations	Ovary	NCT02703571 trial		
31	ERBB2, MYC, PIK3CA amplifications	Ovary	Everolimus + Letrozol		
32	PIK3CA alteration	Cervix	Temsirolimus	Temsirolimus	32
33	PIK3CA and KRAS mutations, MET gene fusion	Cervix	1. Crizotinib 2. Everolimus		
34	KRAS, SMAD4 and PTEN mutations	Endometrium	Everolimus		
35	HTB (27 muts/MB)	Other	1. Checkpoint inhibitor 2. NCT Master trial		
36	EML4-ALK gene fusion	Other	ALK inhibitor		

5.
